# Supplementary material for: Inhibitory Effect of AB-PINACA, Indazole Carboxamide Synthetic Cannabinoid, on Human Major Drug-Metabolizing Enzymes and Transporters
Source: Pharmaceutics. 2020 Oct 29;12(11):1036. doi: 10.3390/pharmaceutics12111036 (PMC7692329; doi:10.3390/pharmaceutics12111036)
Supplement: Supplementary file 1 [file pharmaceutics-12-01036-s001.pdf]

pharmaceutics

Supplementary Materials: Inhibitory Effect of AB-PINACA, Indazole Carboxamide Synthetic Cannabinoid, on Human Major Drug-Metabolizing Enzymes and Transporters

Eun Jeong Park, Ria Park, Ji-Hyeon Jeon, Yong-Yeon Cho, Joo Young Lee, Han Chang Kang, Im-Sook Song and Hye-Suk Lee

1. LC-MS/MS analysis of CYP and UGT metabolites

1.1. LC-MS/MS conditions of eight CYP metabolites in human liver microsomal incubations

Eight CYP metabolites were simultaneously separated on an Atlantis dC18 column (3 μm, 2.1 mm internal diameter × 100 mm; Waters Co., Milford, MA, USA) using a gradient elution of 5% methanol in 0.1% formic acid (mobile phase A, MP A) and 95% methanol in 0.1% formic acid (MP B) at a flow rate of 0.3 mL/min: 20% MP B for 0.5 min; 20–95% MP B for 0.5 min; 95% MP B for 6 min; 95–20% MP B for 0.1 min; and 20% MP B for 3 min. The column and autosampler temperatures were 40 and 4°C, respectively. The electrospray ionization (ESI) source settings in the positive ion mode were: drying gas temperature, 200°C; drying gas flow, 15 L/min; nebulizer pressure, 35 psi; sheath gas temperature, 380°C; sheath gas flow, 11 L/min; capillary voltage, 4,500 V; fragmentor voltage, 380 V; and nozzle voltage, 500 V. Nitrogen gas was used as the collision gas at a pressure of 2 bar on the instrument. Selected reaction monitoring (SRM) transition and collision energy for each CYP metabolite and internal standard (IS) are summarized in Table S1. The data were processed using Mass Hunter software version 7.01 (Agilent Technologies, Wilmington, DE, USA). Representative SRM chromatograms of eight CYP metabolites and two IS are shown in Figure S1.

1.2. LC-MS/MS conditions of six UGT metabolites in human liver microsomal incubations

Six UGT metabolites were simultaneously separated on an Atlantis dC18 column (3 μm, 2.1 mm internal diameter × 100 mm) via gradient elution of 5% acetonitrile in 0.1% formic acid (MP A) and 95% acetonitrile in 0.1% formic acid (MP B) at a flow rate of 0.3 mL/min: 10% MP B for 1 min; 10–50% MP B for 1 min; 50–95% MP B for 1 min; 95% MP B for 2 min; 95–5% MP B for 0.1 min; and 5% MP B for 2.9 min. The ESI source settings in both the positive and negative ion modes were: drying gas temperature, 200°C; drying gas flow, 14 L/min; nebulizer pressure, 40 psi; sheath gas temperature, 380°C; sheath gas flow, 11 L/min; capillary voltage, 4,500 V; fragmentor voltage, 380 V; and nozzle voltage, 500 V. SRM parameters of six UGT metabolites and two IS are summarized in Table S1. The SRM chromatograms of six UGT metabolites and two IS are shown in Figure S2.

2. Supplementary Figures

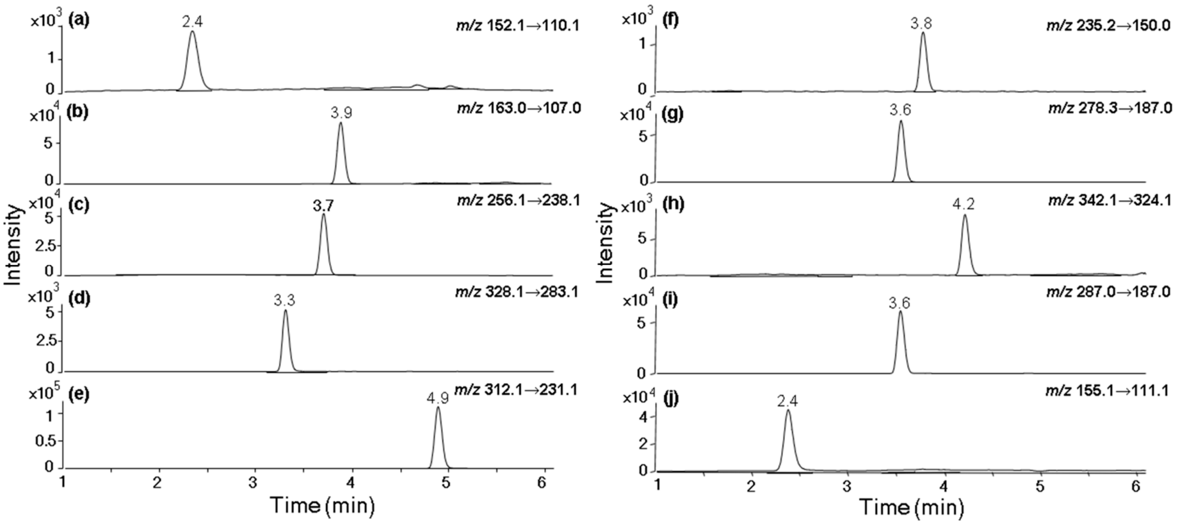

Figure S1. SRM chromatograms of CYP metabolites formed from human liver microsomal incubation of eight CYP cocktail substrates with NADPH and two internal standards (IS). (**a**) acetaminophen, (**b**) 7-hydroxycoumarin, (**c**) hydroxybupropion, (**d**) *N*-desethylamodiaquine, (**e**) 4′-hydroxydiclofenac, (**f**) 4′-hydroxymephenytoin, (**g**) 1′-hydroxybufuralol, (**h**) 1′-hydroxymidazolam, (**i**) d_9-1'-hydroxybufuralol (IS), and (**j**) $^{13}C_2$,^{15}N-acetaminophen (IS).

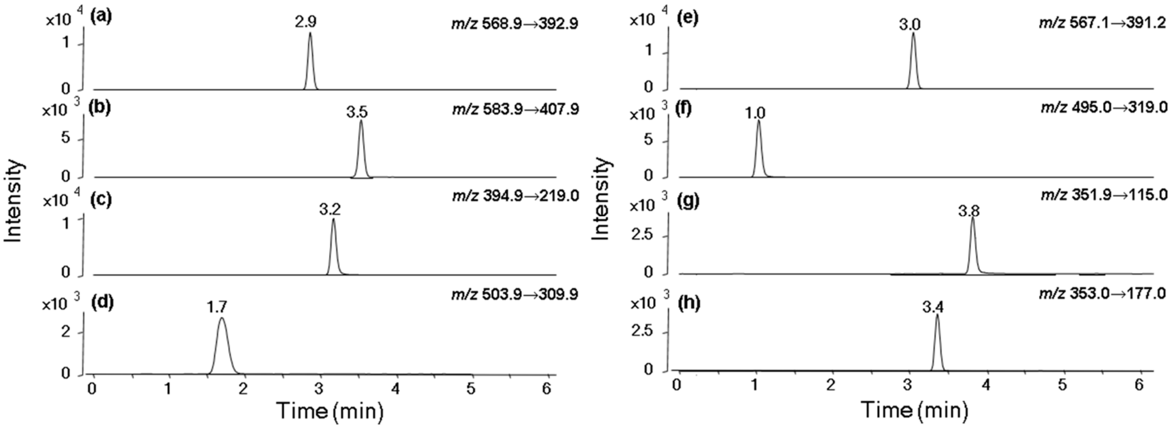

Figure S2. SRM chromatograms of six UGT metabolites formed from human liver microsomal incubation of six UGT cocktail substrates with UDPGA and two IS. (**a**) SN-38 glucuronide, (**b**) chenodeoxycholic acid 24-acyl-β-glucuronide, (**c**) trifluoperazine *N*-glucuronide, (**d**) *N*-acetylserotonin glucuronide, (**e**) mycophenolic acid glucuronide, (**f**) naloxone 3-β-D-glucuronide. (**g**) meloxicam (IS), and (**h**) propofol glucuronide (IS).

3. Supplementary Table

Table S1. SRM parameters of eight CYP metabolites, six UGT metabolites, and their internal standards (IS).

**Analytes**	**SRM transition (*m*/*z*)**	**polarity**	**collision energy (eV)**
CYP metabolites			
acetaminophen	152.1→110.1	ESI^+	8
7-hydroxycoumarin	163.0→107.0	ESI^+	12
hydroxybupropion	256.1→238.0	ESI^+	5
N-desethylamodiaquine	328.1→283.0	ESI^+	55
4′-hydroxydiclofenac	312.0→231.0	ESI^+	17
4′-hydroxymephenytoin	235.2→150.0	ESI^+	8
1′-hydroxybufuralol	278.3→187.0	ESI^+	8
1′-hydroxymidazolam	342.1→324.1	ESI^+	8
d_9-1′-hydroxybufuralol (IS)	287.0→187.0	ESI^+	15
$^{13}C_2$,^{15}N-acetaminophen (IS)	155.1→111.1	ESI^+	8
UGT metabolites			
SN-38 glucuronide	568.9→392.9	ESI^+	30
trifluoperazine *N*-glucuronide	583.9→407.9	ESI^+	26
N-acetylserotonin glucuronide	394.9→219.0	ESI^+	10
naloxone 3-β-D-glucuronide	503.9→309.9	ESI^+	32
chenodeoxycholic acid 24-acyl-β-glucuronide	567.1→391.2	ESI^-	34
mycophenolic acid glucuronide	495.0 →319.0	ESI^-	20
Meloxicam (IS)	351.9→115.0	ESI^+	20
Propofol glucuronide (IS)	353.0 →177.0	ESI^-	28

ESI^+: positive electrospray ionization, ESI^-: negative electrospray ionization.
